# Molecular Xenomonitoring Using Mosquitoes to Map Lymphatic Filariasis after Mass Drug Administration in American Samoa

**DOI:** 10.1371/journal.pntd.0003087

**Published:** 2014-08-14

**Authors:** Mark A. Schmaedick, Amanda L. Koppel, Nils Pilotte, Melissa Torres, Steven A. Williams, Stephen L. Dobson, Patrick J. Lammie, Kimberly Y. Won

**Affiliations:** 1 Division of Community and Natural Resources, American Samoa Community College, Pago Pago, American Samoa; 2 Department of Entomology, College of Agriculture, University of Kentucky, Lexington, Kentucky, United States of America; 3 Department of Biological Sciences, Smith College, Northampton, Massachusetts, United States of America; 4 Division of Parasitic Diseases and Malaria, Centers for Disease Control and Prevention, Atlanta, Georgia, United States of America; Liverpool School of Tropical Medicine, United Kingdom

## Abstract

**Background:**

Mass drug administration (MDA) programs have dramatically reduced lymphatic filariasis (LF) incidence in many areas around the globe, including American Samoa. As infection rates decline and MDA programs end, efficient and sensitive methods for detecting infections are needed to monitor for recrudescence. Molecular methods, collectively termed ‘molecular xenomonitoring,’ can identify parasite DNA or RNA in human blood-feeding mosquitoes. We tested mosquitoes trapped throughout the inhabited islands of American Samoa to identify areas of possible continuing LF transmission after completion of MDA.

**Methodology/Principle Findings:**

Mosquitoes were collected using BG Sentinel traps from most of the villages on American Samoa's largest island, Tutuila, and all major villages on the smaller islands of Aunu'u, Ofu, Olosega, and Ta'u. Real-time PCR was used to detect *Wuchereria bancrofti* DNA in pools of ≤20 mosquitoes, and PoolScreen software was used to infer territory-wide prevalences of *W. bancrofti* DNA in the mosquitoes. *Wuchereria bancrofti* DNA was found in mosquitoes from 16 out of the 27 village areas sampled on Tutuila and Aunu'u islands but none of the five villages on the Manu'a islands of Ofu, Olosega, and Ta'u. The overall 95% confidence interval estimate for *W. bancrofti* DNA prevalence in the LF vector *Ae. polynesiensis* was 0.20–0.39%, and parasite DNA was also detected in pools of *Culex quinquefasciatus*, *Aedes aegypti*, and *Aedes* (*Finlaya*) spp.

**Conclusions/Significance:**

Our results suggest low but widespread prevalence of LF on Tutuila and Aunu'u where 98% of the population resides, but not Ofu, Olosega, and Ta'u islands. Molecular xenomonitoring can help identify areas of possible LF transmission, but its use in the LF elimination program in American Samoa is limited by the need for more efficient mosquito collection methods and a better understanding of the relationship between prevalence of *W. bancrofti* DNA in mosquitoes and infection and transmission rates in humans.

## Author Summary

Lymphatic filariasis (LF), a mosquito-borne parasitic disease, has been targeted for elimination in many countries since the introduction of mass drug administration (MDA) programs using two-drug combinations along with improved diagnostic methods. Sensitive molecular methods detecting parasite DNA in pools of mosquitoes, along with efficient mosquito collection methods, can help identify sites of continuing LF transmission that may require further treatment after MDA has eliminated transmission in most areas. We tested mosquitoes from villages throughout American Samoa after the conclusion of a series of annual MDAs. Widespread but low prevalence of parasite DNA in mosquitoes from two of the five islands suggested continued occurrence of LF. In this study, parasite DNA detection in mosquitoes helped identify areas where human infections exist and additional treatment may be needed. In the future, development of more efficient mosquito collection methods for local species would facilitate larger sample sizes and more precise estimates of prevalence. In addition, developing a better understanding of the epidemiological significance of parasite DNA prevalence in the local mosquitoes will increase the operational value of those estimates for LF elimination programs.

## Introduction

**Figure 1:**
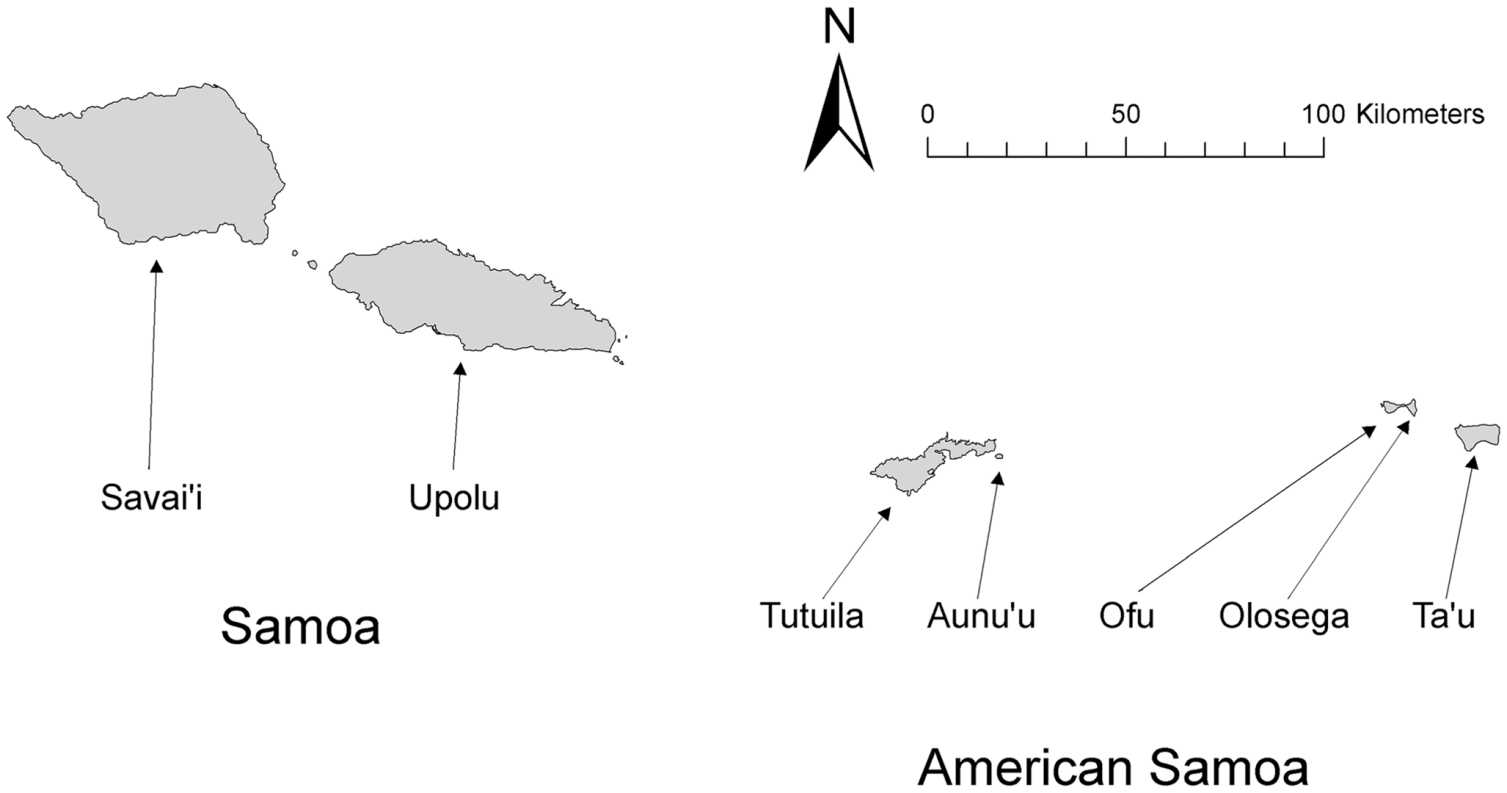
The study was conducted in American Samoa which is composed of the eastern islands of the Samoan Archipelago. (Swains Island and Rose Atoll not shown.)

Lymphatic filariasis (LF) caused by the diurnally subperiodic form of the mosquito-borne parasitic nematode *Wuchereria bancrofti* is endemic to American Samoa, a United States territory composed of the easternmost islands of the Samoan archipelago (Figure 1). LF is also endemic in the archipelago's western islands which comprise the independent nation of Samoa [1], [2]. In the Samoan archipelago, *Aedes* (*Stegomyia*) *polynesiensis* Marks and *Aedes* (*Finlaya*) *samoanus* (Grünberg) are the major vectors of LF [3], [4]. Natural infections have also been detected in *Aedes* (*Stegomyia*) *upolensis* Marks and *Aedes* (*Finlaya*) *tutuilae* Ramalingam and Belkin, but these species are not considered to be as epidemiologically important due to their relatively low abundances in human landing catches [5], [6]. *Aedes polynesiensis* is widespread in the South Pacific, inhabiting islands south of the equator from Tuvalu and Fiji eastward to the Marquesas and Pitcairn Island [7]. It breeds in a wide range of natural and artificial containers [8], [9], [10] and feeds primarily in the daytime [11], [6]. *Aedes polynesiensis* is believed to be a weak disperser, rarely traveling as far as 92 m [12], [11]. *Aedes samoanus* occurs only in American Samoa and Samoa, breeding primarily in water collecting in leaf axils of the forest climber *Freycinetia reineckei* in American Samoa, and in axils of *F. reineckei* and *Pandanus* spp. in Samoa [5], [13]. *Aedes samoanus* females feed at night [5], [6]. The dispersal capabilities of *Ae. samoanus* have not been investigated. Other mosquito species abundant in Samoa and American Samoa are *Culex* (*Culex*) *quinquefasciatus* Say, *Culex* (*Culex*) *annulirostris* Skuse, *Culex* (*Culex*) *sitiens* Wiedemann, *Aedes* (*Stegomyia*) *aegypti* (L.), *Aedes* (*Finlaya*) *oceanicus* Belkin, and *Aedes* (*Aedimorphus*) *nocturnus* (Theobald) [7], [14]; however, none of these species have been found to play a significant role in LF transmission in the Samoan islands [12], [5], [14], [15].

During the years 2000–2010, the American Samoa Department of Health undertook a campaign to eliminate LF through annual mass drug administration (MDA) using diethylcarbamazine and albendazole [16]. The campaign ran in conjunction with similar campaigns in other South Pacific countries and territories, including neighboring Samoa, under the Pacific Programme to Eliminate Lymphatic Filariasis [17]. Population coverage by MDA was 24–52% in the first three years and improved to 65–71% in the subsequent four years [16]. Infection prevalence before, during, and after MDA has been monitored primarily by an immunochromatographic (ICT) test, which detects circulating filarial antigen (CFA) released into the blood by adult *W. bancrofti* [18]. The testing was done across all age groups. Prevalence of CFA in a baseline survey in 1999 was 16.5% [19], and subsequent testing in four sentinel villages found CFA declining from 11.5% in 2001 to 0.95% in 2006 [20]. Prevalences in an additional four villages surveyed in 2006 were higher, ranging from 2.1% to 4.6% [20], [21], and a territory-wide serosurvey in 2007 found 2.3% CFA prevalence. Additional MDA activities took place during 2007–2010, but the level of MDA coverage during those years is unclear.

Testing the human population for CFA can provide information about prevalence of *W. bancrofti* infection, and antibody testing can provide a sensitive indicator of levels of exposure to *W. bancrofti* [22]. In addition, one can sample the human population indirectly by sampling mosquito species known to feed on human blood. Molecular xenomonitoring (MX), the detection of parasite DNA or RNA in mosquitoes using the polymerase chain reaction (PCR), allows the testing of pools of mosquitoes and can be more efficient and more sensitive than dissections, especially when large numbers must be examined to detect evidence of *W. bancrofti* when prevalence is low [23], [24], [25]. The ability to test large numbers of mosquitoes also depends on the availability of efficient collection methods for local species. The development of the BG Sentinel trapping system has, for the first time, made trapping large numbers of *Ae. polynesiensis* over large geographic areas feasible in American Samoa [26].

It is important to recognize that MX cannot provide a direct measurement of ongoing transmission unless the PCR method used specifically targets the infective third stage larva (L3) of *W. bancrofti* [27]. Instead, it provides an indirect assessment of human infection. Fischer et al. [28] and Erickson et al. [29], studying *Brugia malayi*, found that parasite DNA could be detected in both vector and non-vector mosquito species long after ingestion of microfilariae, even when those microfilariae did not survive in the mosquito. Workers wishing to assess transmission directly still need to measure vector biting rates and use dissection or reverse transcriptase-PCR to specifically detect L3 in the vector mosquitoes.

In 2006, a pilot study evaluated the use of MX and traditional xenomonitoring concurrently with serological testing of humans in three villages in American Samoa. Trapped mosquitoes were examined by PCR or dissection, and village residents were tested for CFA and antifilarial antibody [21]. (The Bm14 antibody test used is an indicator of infection or exposure and may give a positive result prior to development of patent infections [30], [31], [32].) The serological tests found 3.7–4.6% of residents of the three villages were positive for CFA and 12.5–14.9% positive for antifilarial IgG4 antibody to the recombinant Bm14 antigen [21]. Dissection of approximately half of the *Ae. polynesiensis* catch found infection prevalences of 0–0.23%, while PCR testing of the remainder gave estimates of 0.52–0.90% prevalence [25]. In summary, mosquito dissection proved relatively insensitive, while antigen and antibody testing and MX all gave similar results. All three indicated LF infections occurring at low levels in all three villages.

In 2011, a territory-wide transmission assessment survey (TAS) was conducted according to the World Health Organization [18] guidelines for monitoring and assessment of MDA in LF elimination programs [33]. The TAS consisted of antigen and antibody testing of 6–7 year olds in the territory's elementary schools. Overall CFA prevalence in the survey was below the threshold at which the guidelines would recommend additional MDA [33]. The TAS results provide guidance to determine whether or not to restart MDA at the territory level. But if LF infection is uneven across subpopulations or across geographic areas, then some groups or areas may require additional MDA even though aggregate LF prevalence is below a level deemed necessary to sustain the infection in the population. The limited dispersal ability of the major LF vector *Ae. polynesiensis* and its susceptibility to the BG Sentinel trap suggested that MX using mosquitoes trapped from throughout American Samoa may be a useful adjunct to the school-based TAS for detecting areas of possible continuing LF transmission. We here describe the results of PCR testing for *W. bancrofti* DNA in mosquitoes captured from villages throughout American Samoa. Results of the TAS will be described elsewhere.

## Methods

### Study area

The mosquito collections were conducted on the islands of Tutuila, Aunu'u, Ofu, Olosega, and Ta'u (Figure 1). These are the only islands in American Samoa that have been continuously inhabited in recent years. The five islands are located between 14° 9′ and 14° 22′S and 169° 25′ and 170° 51′W. The largest, Tutuila Island, comprises 68% of the territory's 199 km^2^ total land area and contains approximately 97% of its total population of 55,519 [34]. Aunu'u Island had 436 residents by the 2010 census [34]. Many of Aunu'u's residents commute by boat to nearby Tutuila for work or school. The more distant Ofu, Olosega, and Ta'u Islands, which together comprise the Manu'a group, had 176, 177, and 790 inhabitants, respectively, according to the 2010 census [34]. Much of the territory's land is forested, steep, and rugged, with about half the area having 70% or greater slope and over half covered by rainforest [35], [36]. Human settlement is mostly along the coastlines, with the exception of the Tafuna-Leone plains and the Aoloau-Aasu uplands areas in the southwest portion of Tutuila Island.

Trapping was conducted within residential areas of all major villages of the four smaller islands and 34 randomly selected villages out of the 67 on Tutuila. These randomly selected villages contained approximately 57% of Tutuila's population and 52% of its land area [34]. In some cases, 2–4 adjacent selected villages on Tutuila Island were combined and treated as single village areas for trapping and analysis. In one case, leaders in a selected village were not available to assist during the trapping time, so a nearby village was used instead.

In the TAS, only two children were identified as CFA positive [33]. These children both attended a school located in a village on Tutuila that was not among those randomly selected for mosquito trapping. As a result, additional trapping was conducted in and around the school grounds using the same procedures as in the selected villages. Because the school was not located in one of the selected villages, data from these traps were not included in the larger data set but are reported separately.

### Mosquito collections

In each village (or group of contiguous smaller villages) ten BG-Sentinel traps baited with BG Lure (Biogents AG, Regensburg, Germany) were placed throughout the village and operated for approximately 24 or 48 h, depending on catch rate. Exceptions occurred in the combined area of Alega and Avaio villages where only six traps were placed, and Amaua village where four traps were placed. Traps were removed after 24 h if it appeared that the catch had reached a target of 200 *Ae. polynesiensis* females. The traps were placed on the ground in locations protected from direct sunlight and rain, often under eaves of houses or outbuildings such as unused open-sided traditional cookhouses. Placements were determined in consultation with village leaders and individual families while attempting to spread the traps evenly throughout the residential area of each village. Although village lands may be extensive, often spanning areas from the coast to the interior ridgetops, in most cases the residential areas are largely confined to lands near the coast or near major roads. Mosquitoes were removed from the traps twice per day at approximately 10:00 am and 6:30 pm following peak feeding times of the major vector *Ae. polynesiensis* [11], [6]. In one village (Vatia) the second trap check scheduled for 10:00 am had to be postponed to 4:30 pm due to a tsunami warning and village evacuation, so the Vatia traps ran for approximately 30.5 h rather than 24 or 48 h. Mosquitoes collected during the first day of trapping in Taputimu and Vailoatai villages were lost, so only the second day's catch was used from these two villages.

In the laboratory, the mosquitoes were anaesthetized with carbon dioxide and identified on a tray resting on an ice pack under a stereomicroscope using the taxonomic keys of Ramalingam [14] and Huang [37]. The few mosquitoes that could not be identified due to damage or that were missing substantial parts of the head, thorax, or abdomen were not included in the analysis. Female mosquitoes were placed in pools of ≤20 (range 1–20) into microcentrifuge tubes separated by species, trap, location, and collection date and time. After freezing to ensure all mosquitoes were dead, the tubes were left open in an oven to dry at 75°C overnight, then closed and stored in a sealed plastic box with dessicant at 23°C until they were shipped for PCR analysis at Smith College, Massachusetts, USA. Trapping was conducted February 21–April 8, 2011 on Tutuila and Aunu'u and June 7–16, 2011 on the more remote Ofu, Olosega, and Ta'u islands.

### DNA extraction from mosquitoes

DNA extraction was done using a modification of the commercial DNeasy kit protocol (Qiagen, Hilden, Germany) and methods adapted from Fischer et al. [38] and Laney et al. [27]. Briefly, a 4.5 mm zinc-plated bead and 180 µl phosphate-buffered saline (pH 7.2) were placed in each round-bottom 2-ml Eppendorf tube (Eppendorf North America, Hauppauge, NY, USA) containing up to 20 dried mosquitoes. The tube was capped and vortexed at high speed in a horizontal position for 15 min and again for an additional 5–10 min if necessary for complete maceration. The tube was centrifuged briefly before adding 20 µl proteinase K and 200 µl of Buffer AL. The mixture was vortexed gently for 3 sec, then incubated at 70°C for 10 min. After brief centrifugation, another 20 µl proteinase K was added and mixed with brief gentle vortexing before incubating for 1 h at 56°C. The mixture was then centrifuged at high speed, and the supernatant from each tube was added to a 1.5-ml Eppendorf tube containing 200 µl of 95–98% ethanol and mixed using the pipet. The entire mixture from each tube was then applied to a DNeasy kit column and centrifuged at 8,000 *g* for 1 min. The column was transferred to another 1.5-ml tube, and the DNA was washed twice with 500 µl of Buffer AW1, with each wash followed by a 1 min centrifugation at 8,000 *g*. The column was then transferred to another 1.5 ml tube, 500 µl Buffer AW2 was added, and the tube spun at 8,000 *g* for 3 min. The waste solution was discarded, and the column spun an additional 3 min at maximum speed to dry the column. The column was then transferred to a 1.5-ml microfuge tube and the DNA was eluted twice with 125 µl of Buffer AE followed by 2 min centrifugation, first at 8,000 *g*, and then at 10,000 *g*. The samples were held at 4°C until the qPCR was completed, then stored at −20°C.

### qPCR detection of *W. bancrofti* DNA

Real-time PCR was done using a 7300 Real-Time PCR System (Applied Biosystems, Foster City, California, USA). Each reaction contained 1 µl of template DNA and 24 µl of qPCR master mix including 10 µM each of forward and reverse primers and taqman probe. The primers were designed to amplify a fragment of the “long dispersed repeat” of *W. bancrofti* (LDR; GenBank accession no. AY297458) [39]. The sequence of the primers and probe were as follows [39]: forward primer (Wb-LDR1) 5′-ATTTTGATCATCTGGGAACGTTAATA-3′, reverse primer (Wb-LDR2) 5′-CGACTGTCTAATCCATTCAGAGTGA-3′, and probe (Wb-LDR) 6FAM-ATCTGCCCATAGAAATAACTACGGTGGATCTCTG-TAMRA. The cycling conditions were 50°C for 2 min and 95°C for 10 min, followed by 40 cycles of 95°C for 15 sec and 60°C for 1 min. Four different controls were used: a negative extract control consisting of a DNA extract from 20 uninfected mosquitoes; positive PCR controls using 1 ng, 100 pg, or 10 pg DNA of *W. bancrofti*; a negative PCR control using the same ddH_2_O as used in the master mix; and a PCR inhibitor control comprised of 5 pg of *W. bancrofti* DNA added to 10 µl of negative extract control. The negative extract and PCR inhibitor controls were run periodically throughout the course of sample processing. Positive and negative PCR controls were run with every sample batch. Samples were run in duplicate, and qPCR results with Ct≥39 were checked by running two additional qPCR reactions on the same extract template. If the sample was positive at least once more, and all controls were as expected, then the sample was considered positive. If both verification reactions were negative, then the sample was considered negative.

### Statistical analysis

Geographic coordinates were recorded for each trap location using a Trimble GeoXT 2005 Series Pocket PC handheld global positioning system (GPS) device (Trimble Navigation Ltd., Sunnyvale, California, USA). For 16 out of the 310 trap locations, the Trimble device was unable to record the positions due to topography, tree cover, weather conditions or satellite positions at the time, so a Garmin GPSmap 60CSx (Garmin International, Inc., Olathe, Kansas, USA) device was used instead for those locations. The positions were mapped using ArcGIS 10.1 software (Environmental Services Research Incorporated, Redlands, California, USA), and village boundaries were obtained from the 2010 U.S. Census Bureau's TIGER/Line “Places” shapefile for American Samoa [40]. In a few cases, traps were placed in locations which were inside the village boundaries as indicated by village leaders, but which fell outside the boundaries on the Census Bureau map. Point estimates and 95% confidence intervals for the percentage of mosquitoes containing *W. bancrofti* DNA were calculated for each mosquito species for the overall sample and for the most abundant species, *Ae. polynesiensis*, within each of the villages. The program PoolScreen (version 2.0.3) was used to calculate maximum likelihood point estimates of prevalence, and confidence intervals were determined by the likelihood ratio method [41].

## Results

**Figure 2:**
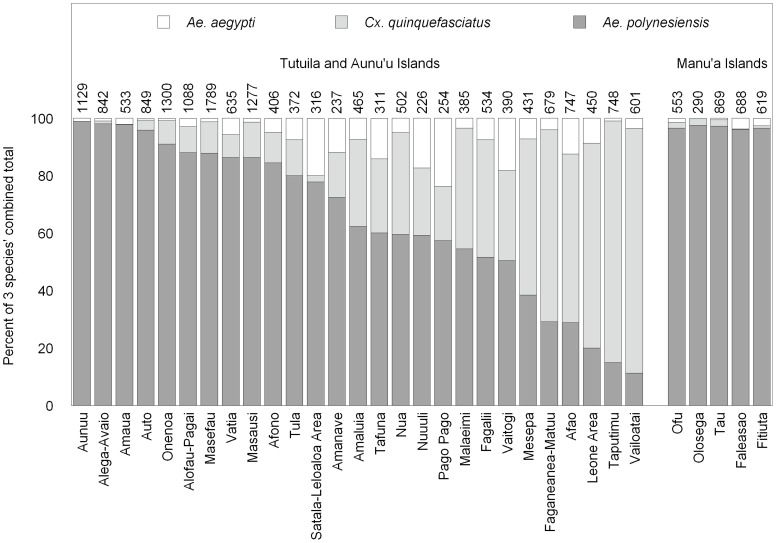
Catch of the three most numerous mosquito species which had >1 positive pool overall as a percentage of those three species' combined total in each village. The number above each bar is the combined total number captured of the three species. Ten traps were operated for 1–2 days in each village, except in Alega-Avaio and Amaua in which six and four traps were used, respectively. “Satala-Leloaloa Area” includes Satala, Anua, Atuu, and Leloaloa villages and “Leone Area” includes Auma, Leone, and Puapua villages.

A total of 22,014 female mosquitoes were collected and sorted into 2,629 pools of ≤20 individuals each for PCR testing. PCR results for the most abundant species in the traps are shown in Table 1, and relative abundances of the three most numerous species having >1 positive pool are shown in Figure 2. Members of the *Aedes* (*Finlaya*) group of species occurring in American Samoa include *Ae. oceanicus*, *Ae. samoanus*, and *Ae. tutuilae*. They were difficult to distinguish due to their morphological similarity and the loss of scales in the traps, so were combined for PCR testing and analysis. Only one out of the 267 pools of *Ae.* (*Finlaya*) spp. was positive by PCR. Other species captured in lower numbers were *Ae. nocturnus*, *Cx. annulirostris*, and *Cx sitiens*. *Wuchereria bancrofti* DNA was not detected in these species (n = 68 pools). *Aedes polynesiensis*, *Cx. quinquefasciatus*, *Ae. aegypti*, and *Ae.* (*Finlaya*) group species all produced positive pools (Table 1). Estimated prevalence was highest in *Ae. aegypti*, although the 95% confidence interval for prevalence in this species overlapped with that for *Ae. polynesiensis* (Table 1).

**Table 1:** Detection of *W. bancrofti* DNA in American Samoa mosquitoes by PCR.

Species	Females	Pools^1^	Positive Pools	Prevalence^2^	95% Confidence Interval^3^
*Ae. polynesiensis*	15,215	1,250	42	0.28%	0.20, 0.39%
*Cx. quinquefasciatus*	4,413	585	5	0.11%	0.034, 0.27%
*Ae. aegypti*	887	360	8	0.92%	0.37, 1.8%
*Ae.* (*Finlaya*) spp.^4^	1,084	267	1	0.092%	0.0028, 0.48%
*Ae. upolensis*	262	91	0	0%	0, 0.73%

1 Pools were comprised of ≤20 females.

2 Prevalence estimate by maximum likelihood.

3 Confidence intervals by likelihood ratio method. (One-sided when prevalence estimate is 0.).

4 May include *Ae. oceanicus*, *Ae. samoanus*, *Ae. tutuilae*.

**Figure 3:**
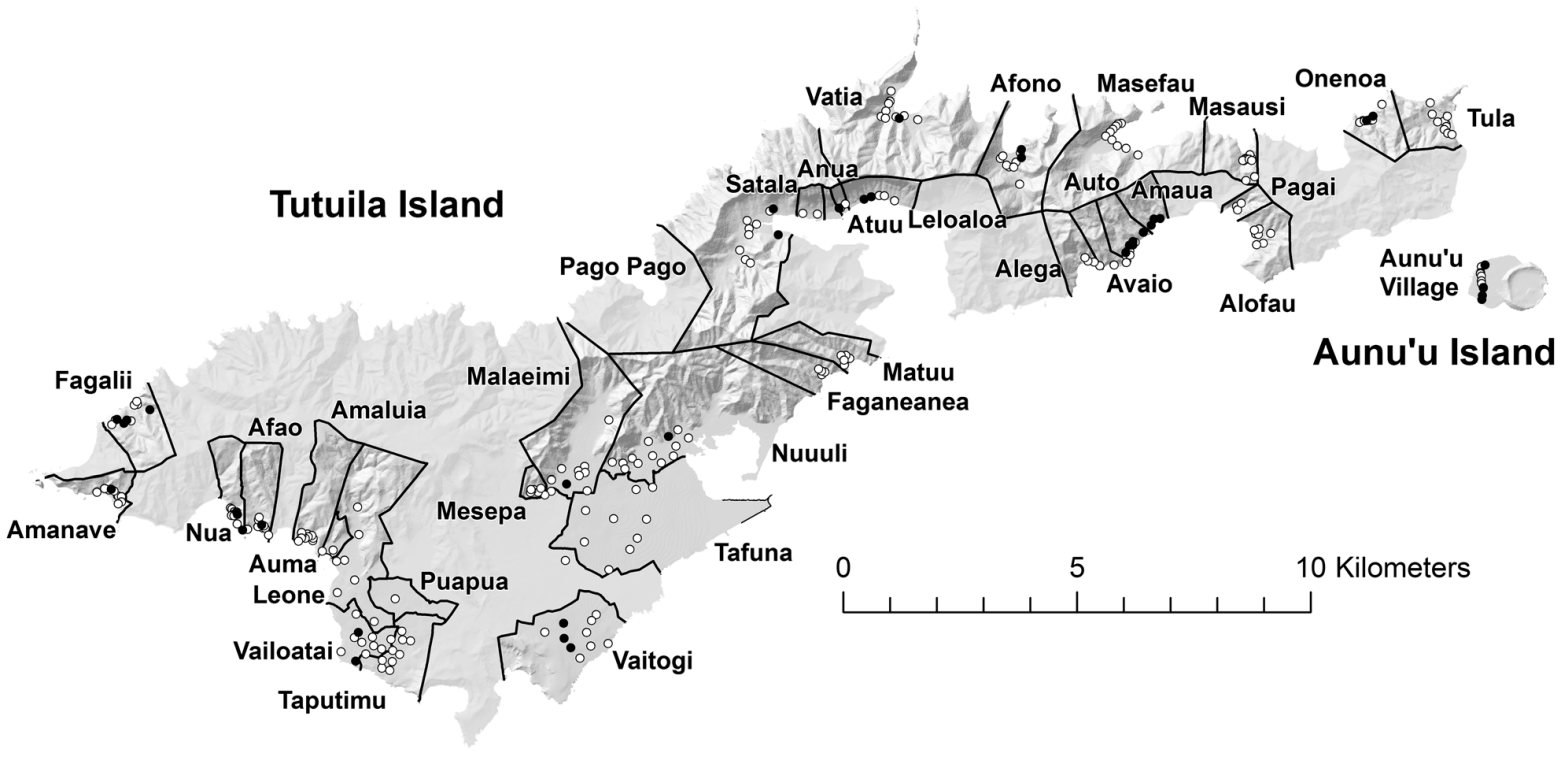
Mosquito trapping locations in villages on Tutuila and Aunu'u Islands, American Samoa. Filled circles represent traps which captured mosquitoes in which PCR testing detected *W. bancrofti* DNA.

**Figure 4:**
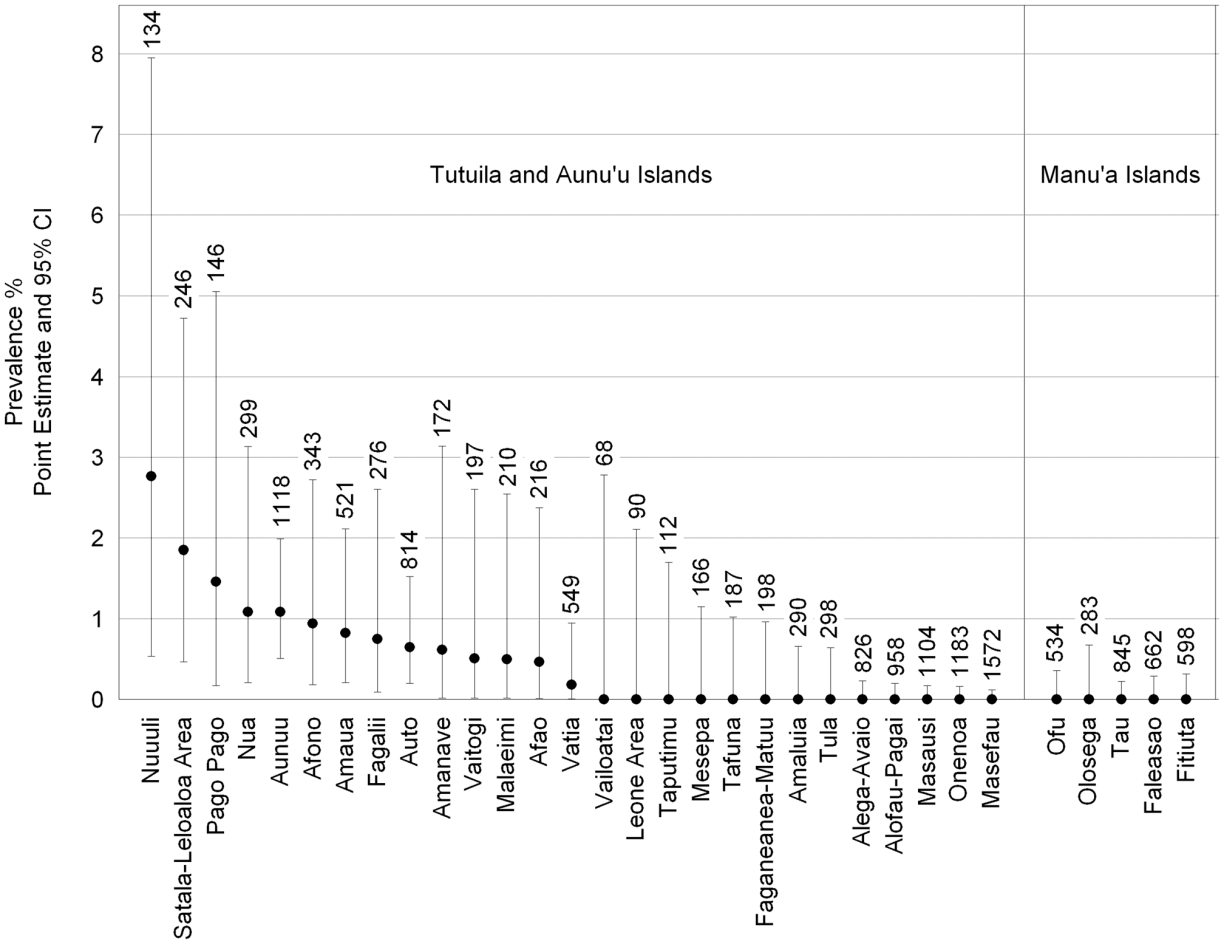
Estimated prevalence of *Ae. polynesiensis* females containing *W. bancrofti* DNA from trap catch in each village. Prevalences were estimated by maximum likelihood and confidence intervals by the likelihood ratio method [41]. The total number of *Ae. polynesiensis* is shown above each bar. “Satala-Leloaloa Area” includes Satala, Anua, Atuu, and Leloaloa villages and “Leone Area” includes Auma, Leone, and Puapua villages.

**Figure 5:**
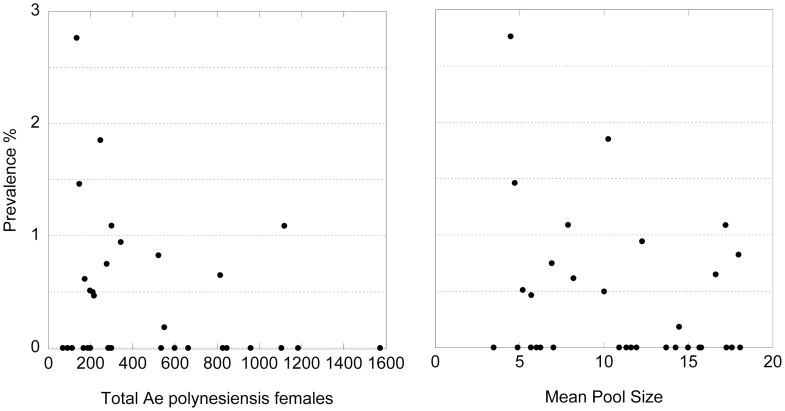
Estimated prevalence of *Ae. polynesiensis* containing *W. bancrofti* DNA for each village versus total *Ae. polynesiensis* females tested (left) and mean pool size (right).

There were no positive pools of any species collected from the five major villages of the Manu'a Islands of Ofu, Olosega, and Ta'u. For *Ae. polynesiensis*, the most abundant species captured there, the upper limit for the one-sided 95% confidence interval estimate of prevalence across all three Manu'a Islands was 0.066% (n = 212 pools). On Tutuila and Aunu'u islands, 38 out of 260 total trap placements produced at least one positive pool. Positive mosquitoes were detected in the majority (16 out of 27) of the village areas sampled on these two islands. Areas producing positive mosquitoes on Tutuila Island were widely distributed throughout the island (Figure 3). *Aedes polynesiensis* was by far the most abundant mosquito species trapped overall, and prevalence estimates for *Ae. polynesiensis* from the villages are depicted in Figure 4. There was no evidence of a positive relationship between prevalence estimate and number of *Ae. polynesiensis* females or mean pool size (Figure 5), suggesting that the number of mosquitoes collected affected the breadth of confidence intervals as evident in Figure 4, but not prevalence point estimates. Nine traps which produced no positive pools of *Ae. polynesiensis* did produce positive pools of *Cx. quinquefasciatus* (5 traps), *Ae. aegypti* (6 traps), or *Ae.* (*Finlaya*) spp. (1 trap). At the village level, two villages with no positive *Ae. polynesiensis* catches had positive *Cx. quinquefasciatus* (Onenoa and Vailoatai) or *Ae. aegypti* (Vailoatai) pools.

Of the ten traps placed in and around the grounds of the elementary school attended by two children who tested positive for CFA in the TAS, five of the traps produced positive mosquito pools. Two of these traps had positive *Ae. polynesiensis*, two had positive *Ae. aegypti*, and one trap had both positive *Ae. polynesiensis* and positive *Ae. aegypti*. Prevalence estimates were 2.8% with a 95% confidence interval of (0.55–8.0%) (n = 107 females) for *Ae. polynesiensis* and 8.6% with a 95% confidence interval of (2.2–20.8%) (n = 55 females) for *Ae. aegypti*. Pools of the 84 *Cx. quinquefasciatus* and four *Ae.* (*Finlaya*) spp. females collected around the school were all negative.

## Discussion

Molecular xenomonitoring of mosquitoes trapped from villages throughout American Samoa found evidence of low but widespread occurrence of *W. bancrofti* infections on Tutuila and Aunu'u islands which together are home to 98% of the territory's population. The study did not find evidence of infections on Ofu, Olosega, and Ta'u islands. The ability to detect very low *W. bancrofti* prevalences was limited, however, due to the low numbers of mosquitoes collected in many of the villages. This lack of sensitivity was reflected in the wide confidence intervals on prevalence estimates for many of the villages (Figure 4). Mosquito collection efforts and the number of pools that could be tested were limited by the resources available for the project.

The type of mosquito collection method used may also have affected the sensitivity of xenomonitoring [42]. Female mosquitoes can contain *W. bancrofti* DNA only after they have completed at least one blood meal. The BG Sentinel traps used in this study are designed to capture host-seeking females, many of which may be nullipars seeking their first blood meal. Collections with gravid traps targeting ovipositing females [43], [44] can help ensure that a larger portion of the mosquitoes captured will have had at least one blood meal, but currently available gravid traps catch few *Ae. polynesiensis* (MAS unpublished data). For endophagic species, collection of resting mosquitoes in houses can also produce larger proportions of previously blood-fed females [45], [24]. Gravid traps and collection of resting mosquitoes in houses have been effective for *Cx. quinquefasciatus* xenomonitoring in areas where that species is the major LF vector. *Culex quinquefasciatus* does not appear to be an important LF vector in the Samoan islands [15], but it was the second most abundant species in our BG Sentinel traps and an estimated 0.11% contained *W. bancrofti* DNA. In villages where this species is abundant (Figure 2), use of gravid traps targeting *Cx. quinquefasciatus* in place of, or in addition to, BG Sentinel traps targeting *Ae. polynesiensis* might improve xenomonitoring efficiency by increasing both the capture rate and the proportion of the catch consisting of previously blood-fed individuals. This approach remains to be tested in American Samoa.

The large proportion of traps which produced positive mosquitoes in the area of the school at which two children tested positive for CFA indicated possible ongoing transmission there. Examination of blood smears and PCR testing following the ICT failed to find evidence of microfilaremia in either child [33], suggesting they may not have been the sources of the *W. bancrofti* detected in the trapped mosquitoes. The two children came from different villages, and each lived approximately 1 km from the school. Because *Ae. polynesiensis* feeding times overlap with times when students are at school and at home [11], [6], transmission by this vector could occur in either setting.

According to the 2010 census [46], approximately 21,196 of American Samoa's population attended school (pre-kindergarten – college) and 12,070 of the territory's 16,482 working population traveled more than 15 min from home to work. The mobility of the human population and the daytime feeding habits of *Ae. polynesiensis* suggest that *W. bancrofti* transmission likely occurs not only in residential areas of villages, but also at other locations, such as workplaces, bus stops, and schools. With the exception of the single school, this study did not sample these other potentially important locations.

There were several similarities between the results of this study and the only other study to use MX in American Samoa [25]. Only one of the three villages sampled by Chambers et al. [25] was sampled again in the current study. Prevalence of *W. bancrofti* DNA in *Ae. polynesiensis* for Afao Village was estimated to be 0.82% in the 2006 study and 0.47% in the current one. The wide confidence intervals obtained in the two studies (Figure 4 here and Figure 4 of Chambers et al. [25]) indicate a much larger sample size would be required to evaluate the significance of a difference of this magnitude. The estimates for prevalence of *W. bancrofti* DNA in *Ae. aegypti* were higher than those for *Ae. polynesiensis* both in this study and in the 2006 study, although the 95% confidence intervals for the two species overlapped broadly in both cases. The high propensity of *Ae. aegypti* for feeding on human hosts is well documented (e.g., [47], [48]) and could result in a higher frequency of feeding on microfilaraemic individuals than would be the case for mosquito species with less affinity for humans. *Aedes polynesiensis* is known to feed on birds and mammals other than humans, but little is known about the frequency with which it feeds on the different hosts [11], [49], [5]. No *W. bancrofti* DNA was detected in the 262 *Ae. upolensis* collected from throughout the territory in the current study. A similar number of *Ae. upolensis* collected from three villages in the earlier study by Chambers et al. [25] produced one positive pool. The low incidence of *W. bancrofti* DNA in this species and the low numbers collected in villages support the suggestion that it is likely a minor vector of LF in American Samoa [14].

Positive PCR results for species not considered to be important LF vectors revealed evidence of *W. bancrofti* in some locations where results from *Ae. polynesiensis* collections did not. Only two of the six traps with positive pools of *Ae. aegypti* and only one of the five traps with positive *Cx. quinquefasciatus* also produced positive *Ae. polynesiensis*. At the village level, two villages (Onenoa and Vailoatai) produced positive *Ae. aegypti* or *Cx. quinquefasciatus* pools from multiple traps, but no positive *Ae. polynesiensis* pools. The discrepancies are likely due to behavioral differences and variation in relative abundance of the three species across trapping sites. Together they suggest that sampling multiple species—including non-vectors—with different feeding behaviors may provide a more complete assessment of *W. bancrofti* infections than sampling only a single important vector species. The three species exhibit important differences in feeding behavior [50], [7], [5]. *Aedes aegypti*, like *Ae. polynesiensis*, feeds primarily during the day, but is more endophilic than *Ae. polynesiensis*. *Culex quinquefasciatus* feeds mainly at night and feeds and rests both inside and outside houses. Differences in range of movement could also result in different exposures to *W. bancrofti*. *Aedes aegypti* and *Ae. polynesiensis* are believed to have limited dispersal ability [12], [11], [51], but *Cx. quinquefasciatus* may move longer distances [52], [53], [54], [55]. Finally, if multiple species are included in xenomonitoring, the reduced sensitivity resulting from a low catch rate for *Ae. polynesiensis* in some villages, as occurred in Vailoatai, might be partially compensated for by higher catches of other species (Figure 2).

Xenomonitoring using multiple species, including non-vectors, is a departure from the approach of monitoring only a single vector species and comparing estimated prevalence in that species to model-based or empirical thresholds to assess progress in LF elimination programs [24], [42]. The latter approach is complicated in the Samoan islands due to the presence of an important secondary vector, *Ae. samoanus*, the lack of an effective trap for that species, and the difficulty in distinguishing it morphologically from a closely related non-vector species. Another complication is the spatial heterogeneity of LF prevalence and transmission [56], [57] which suggests that even when aggregate prevalence in mosquitoes captured over a large area may fall below a target threshold, some local prevalences may exceed it. In addition, earlier xenomonitoring efforts have revealed that *W. bancrofti* prevalence in *Ae. polynesiensis* collected at a single location can vary substantially over the course of a year or even between collection periods separated by as few as ten days [58], [25]. Together, these factors, along with the difficulty of collecting large numbers of vectors and the resulting wide confidence interval estimates, suggest that xenomonitoring currently has limited usefulness for quantifying the progress of LF elimination in American Samoa. Instead its operational value may lie in helping to map areas where human infections exist without the invasiveness of human blood collection. Even such presence-absence mapping, however, requires trapping sufficient mosquitoes at each location to provide a high probability of detecting positive mosquitoes in the locations where they occur—something that may be difficult to achieve in areas where prevalence and catch rates are low.

In summary, the detection of *W. bancrofti* DNA in mosquitoes at many locations on Tutuila and Aunu'u islands suggests widespread occurrence of human infections on these islands, while the low overall prevalence estimate suggests a similarly low overall prevalence of human infections. But caution is required in making inferences about prevalence at more local levels due to small sample sizes in many villages. Currently xenomonitoring has little value for programmatic decision-making in American Samoa beyond its ability to identify areas where human infections may exist. Increasing its relevance to MDA decision-making will require additional research to develop more efficient mosquito collection methods and to improve understanding of the relationship between prevalence of *W. bancrofti* DNA in mosquitoes, infection rates in humans, and resulting transmission rates relative to critical thresholds.
